# Point-of-care ultrasonography: a practical step in the path to precision in critical care

**DOI:** 10.1186/s13054-017-1802-2

**Published:** 2017-08-16

**Authors:** Gentle Sunder Shrestha

**Affiliations:** Department of Anaesthesiology, Critical Care Unit, Tribhuvan University Teaching Hospital, Maharajgunj, Kathmandu, Nepal

Management of critically ill patients is challenged by the complexity of underlying pathophysiology, ambiguity of illness syndromes, and rapidly changing physiological status. These unique characteristics of critical illness also justify the need for precision medicine, rather than applying a “one-size-fits all” approach in the ICU [1]. Many large clinical trials, even though supported by strong pre-clinical and pathopysiological bases, have failed to show clinical benefit, probably due to enrollment of heterogeneous patient populations and failure to identify endotypes [2].

Although it is an intriguing concept and there is a need for it, multiple challenges need to be overcome before the application of precision medicine in the ICU in clinical practice [1]. Designing future trials to test individualized therapy, developing and validating biomarkers, and analysis and interpretation of big data are time-consuming and expensive and may not be practical.

For a test or investigation to be practically applicable in the ICU, it should be applicable at the point-of-care, inexpensive, simple, and be feasible and repeatable at the bedside with a rapid turnover time. Critical care echocardiography (CCE) has been shown to be a valuable non-invasive bedside tool for the management of shock. It helps in the rapid evaluation of physiological derangement in patients with shock, to guide therapy, and to evaluate the response to treatment in real time [3]. When combined with critical care ultrasonography (CCUS), CCE is beneficial for rapid evaluation of acute respiratory failure, for adjusting ventilator setting and PEEP titration in ARDS, for identification of patients at risk for weaning failure, for diagnosis of pleural and lung pathologies, and thus for individualize patient management at the bedside [4].

International statements on training standards for CCUS recommend basic CCE and general CCUS to be essential requirements of ICU training programs. CCUS is suggested to be the primary point-of-care imaging modality in the ICU. With the availability of cheaper, compact, and portable ultrasound units, its potential benefit and applicability seem greater and more pragmatic considering the cost and lack of imaging modalities like CT or MRI in resource-poor settings. The approach of whole-body point-of-care ultrasonography (POCUS) can be a practical and viable step in the path to precision in the ICU. The findings of bedside whole-body POCUS in a non-organ-specific manner are integrated with the findings of overall clinical assessment of the patient to individualize patient management [5].

## Abbreviations

ARDS: Acute respiratory distress syndrome
CCE: Critical care echocardiography
CCUS: Critical care ultrasonography
CT: Computerized tomography
ICU: Intensive care unit
MRI: Magnetic resonance imaging
PEEP: Positive end expiratory pressure
POCUS: Point-of-care ultrasonography
